# Stereotactic LINAC-Radiosurgery for Glomus Jugulare Tumors: A Long-Term Follow-Up of 27 Patients

**DOI:** 10.1371/journal.pone.0129057

**Published:** 2015-06-12

**Authors:** Faycal El Majdoub, Stefan Hunsche, Alhadi Igressa, Martin Kocher, Volker Sturm, Mohammad Maarouf

**Affiliations:** 1 Department of Stereotaxy and Functional Neurosurgery, University Hospital of Cologne, Cologne, Germany; 2 Department of Stereotaxy and Functional Neurosurgery, University of Witten-Herdecke, Cologne-Merheim Medical Center (CMMC), Cologne, Germany; 3 Department of Neurosurgery, University of Witten-Herdecke, Cologne-Merheim Medical Center (CMMC), Cologne, Germany; 4 Department of Radiation Oncology, University Hospital of Cologne, Cologne, Germany; 5 Department of Neurosurgery, University Hospital of Wurzburg, Wurzburg, Germany; Uppsala University, SWEDEN

## Abstract

**Background:**

The optimal treatment of glomus jugulare tumors (GJTs) remains controversial. Due to the critical location, microsurgery still provides high treatment-related morbidity and a decreased quality of life. Thus, we performed stereotactical radiosurgery (SRS) for the treatment of GJTs and evaluated the long-term outcome.

**Methods:**

Between 1991 and 2011, 32 patients with GJTs underwent SRS using a linear accelerator (LINAC) either as primary or salvage therapy. Twenty-seven patients (median age 59.9 years, range 28.7–79.9 years) with a follow-up greater than five years (median 11 years, range 5.3–22.1 years) were selected for retrospective analysis. The median therapeutic single dose applied to the tumor surface was 15 Gy (range 11–20 Gy) and the median tumor volume was 9.5 ml (range 2.8–51 ml).

**Results:**

Following LINAC-SRS, 10 of 27 patients showed a significant improvement of their previous neurological complaints, whereas 12 patients remained unchanged. Five patients died during follow-up due to old age or other, not treatment-related reasons. MR-imaging showed a partial remission in 12 and a stable disease in 15 patients. No tumor progression was observed. The actuarial overall survival rates after five, ten and 20 years were 100%, 95.2% and 79.4%, respectively.

**Conclusions:**

Stereotactic LINAC-Radiosurgery can achieve an excellent long-term tumor control beside a low rate of morbidity in the treatment of GJTs. It should be considered as an alternative therapy regime to surgical resection or fractionated external beam radiation either as primary, adjuvant or salvage therapy.

## Introduction

Glomus bodies, firstly described by Stacy Rufus Guild in 1941, are part of the chemoreceptor system and composed of non-chromaffin epitheloid cells surrounded by a thin fibrous capsule embedded in a capillary network [1, 2].

So-called glomus jugulare tumors (GJTs), also called paragangliomas or chemodectomas have their epicenter within the adventitia of the jugular bulb and arise in the chemoreceptoric extra-chromaffin paraganglia. These tumors are rare with an estimated incidence of 1 per 1 million people and are highly vascularized in nature [3, 4]. Usually, GJTs are benign, slow growing but locally aggressive with infiltration of the adjacent bone as well as compression of the brainstem and nervous structures. In about 3% of patients the tumor is considered as malignant with metastatic potential [5, 6]. A typical manifestation time is between the third and sixth decade of life with a significant predominance of the female gender [1]. Multiple GJTs are reported in nearly 10% of cases and a familial form with an autosomal dominant inheritance pattern has a special predilection for tumor multiplicity [7, 8]. Due to the destructive growth, GJTs may expand intracranially leading to symptoms depending on their extension. Most of the patients present with pulsatile tinnitus, conductive hearing loss, dizziness and dysfunction of the cranial nerves V, VII and IX-XII. Involvement of the dural sinuses may mimic sinus thrombosis [9].

Microsurgical complete resection is often complicated and carries a high risk of morbidity or even mortality in some cases due to the high vascularization and proximity to vascular and nervous structures [1, 10, 11]. In experienced hands, microsurgery can achieve high progression-free survival rates, but otherwise also high rates of new cranial nerve palsies [7, 12, 13]. Therefore, a surgical treatment is indicated in cases of intracranial hypertension such as brainstem compression.

Embolization alone cannot prevent tumor progression and is usually performed preoperatively to reduce possible blood loss during surgery.

Several studies of fractionated radiotherapy for GJTs have been published showing local tumor control rates over 90% after 10 years with simultaneously low morbidity rates [14–16]. It is important to note, that the goal of radiation-based therapies is disease control achieved through growth inhibition rather than tumor elimination.

Radiosurgery evolved into an important postoperative adjunct or even into a possible alternative to microsurgery [1, 10, 11, 17]. To date, the number of reports on the use of radiosurgery for the treatment of GJTs has increased.

In this study, we present a single-institution long-term follow-up of 27 patients harboring from GJTs treated with stereotactically guided radiosurgery using a linear accelerator (LINAC-SRS).

## Methods

### Patients, ethics statement and study design

We performed a retrospective analysis of 27 patients suffering from glomus jugulare tumors grades D_1_ or D_2_ according to the classification of Fisch (Table 1) [18].

**Table 1:** Classification of Glomus jugulare tumours according to Fisch^18^.

A	Limited to glomus tympanon
B	Limited to tympanomastoid area with/without erosion of jugular bulb
C	Involvement and destruction of infralabyrinthine and apical compartments
D_1_	Intracranial extension < 2 cm in greatest diameter
D_2_	Intracranial extension > 2 cm in greatest diameter
D_3_	Inoperable intracranial invasion

No separate ethics application and statement by the ethical committee for this retrospective study are required. This study has been evaluated in accordance with German data protection legislation (S1 File). This, in particular, means that the results of the study have been obtained in a completely anonymous manner. The authors FE, MK, VS and MM as well as the referring physicians had contact to patients and access to patient’s data during medical treatment and follow-up evaluations. For all kinds of treatment done at the Department of Stereotaxy and Functional Neurosurgery Cologne it is mandatory to obtain written informed consent of patients scheduled for treatment. In case of minors, this consent is granted either by their parents or by a court-approved caregiver.

Already in 2003 we published the intermediate-term results of 12 patients with GJTs treated between 1991 and 1999 with a median follow-up time of four years [17]. Up to December 2011, 32 patients were treated and 27 patients with a minimum follow-up time of over five years (median 11 years, mean 12.3 years, range 5.3–22.1 years) were evaluated in terms of tumor response, neurological outcome and treatment-related side effects including potential radiation-induced malignancies. Patient characteristics are displayed in Table 2.

**Table 2:** Patient characteristics and treatment parameters.

Patients (n)	27		
Gender (m/f)	5/22		
	median	mean	range
Age (years)	59.9	57.2	28.7–79.9
KPS	80	81	60–90
Follow-up (months)	129.2	148	64–266
Tumor surface dose (Gy)	15	14.8	11–20
Maximum dose (Gy)	18.8	20.3	15–33.9
Isodose level (%)	80	74.3	44–92
Coverage (%)	98.1	96.7	83.5–99.9
VOI 10 (ml)^1^	1.7	2.8	0.4–10.6
D 95Vol% (Gy)^2^	15.7	16	12.7–24.2

Abbreviations: KPS, Karnofsky performance score; Gy, Gray; ml, milliliter

^1^VOI 10: Volume of the peritumoral healthy brain tissue receiving at least 10 Gy^29^

^2^D 95: 95% of the target volume receiving the tumor surface dose

The median time from first diagnosis to LINAC-SRS was 16.4 months (mean 34.8 months, range 2.1–150.7 months). Thirteen patients were treated primarily while 12 were treated for growing tumor remnants after subtotal microsurgical resection or recurrent tumors after total microsurgical resection at different institutions. Two patients were treated for continued tumor growth after embolization. Adjuvantly to subtotal microsurgical resection, one patient received external beam irradiation (EBI) with a total dose of 64 Gy. The most common symptoms from GJTs are displayed in Table 3.

**Table 3:** Patient symptoms preoperatively and improvement after treatment.

Symptoms	Prior LINAC-RS	Improvement after LINAC-RS
Hyp-/Anacusis	17	2/17 (11.8%)
Pulsatile tinnitus	15	6/15 (40%)
Weakness of cranial nerves V, VII, IX-XII	13	5/13 (38.5%)
Vertigo/dizziness	11	6/11 (54.5%)
Cephalgia	6	5/6 (83.3%)
Otalgia	4	3/4 (75%)

### Procedure and technical data

All patients underwent enhanced stereotactical computed tomography (CCT) and cranial magnetic resonance imaging (cMRI) as a basis for stereotactic planning. The patient characteristics and dosimetric treatment parameters are displayed in Table 2.

Inclusion criteria for LINAC-SRS were tumor spread or tumor size (multiple localization and maximum diameter >4 cm in CCT and/or cMRI means exclusion for LINAC-SRS), no considerable brainstem compression or signs of increased intracranial pressure, Karnofsky Performance Score (KPS) ≥60 and patient refusal to microsurgery.

Radiosurgery was performed with a linear accelerator (SL25, ELEKTA, 6 MeV photon beams) adapted for stereotactic radiosurgery and endued with changeable cylindric collimators (3–30 mm in diameter openings) or, since March 2001, a computer-controlled micro multi-leaf collimator (μMLC, 1.5 mm lamella width, maximum field size 72x68 mm, Siemens, Heidelberg, Germany). The patients were fixed under local anesthesia in a modified Riechert-Mundinger stereotactic frame [19]. To enhance the tumor and to visualize blood vessels for landmark correlation 100 ml of contrast medium was applied 15–30 minutes prior and another 40–80 ml directly before CCT scanning, respectively. On these stereotactic CCT scans (slice thickness 1.25–2 mm) and fused cMR-images (contrast enhanced axial T_1_- and T_2_-weighted sequences, slice thickness 1.5 mm performed 1–3 days before treatment) the borders of the tumor were delineated. For each target volume up to 20 beams were applied to match the tumor shape in order to achieve a highly conformal dose distribution. For cylindric collimator planning we used the STP 3.5 software (until February 1996 STP 2.0, Leibinger, Freiburg, Germany) and for μMLC planning VIRTUOSO 3.0 (Stryker-Leibinger, Freiburg, Germany).

### Follow-up

We obtained the clinical follow-up data either from each patient or from their referring physicians. For the radiological follow-up we requested the first cMRI after treatment at six months and at one year thereafter.

Tumor response was evaluated by volumetric measurements and classified according to the Macdonald criteria [20].

### Statistical analysis

The statistical analysis was performed with PASW Statistics 22 (SPSS Inc., IL, USA). The baseline was the date of LINAC-SRS and the endpoints of evaluation were death of the patient despite any cause or the last contact to the patient. The Wilcoxon test was used to compare non-normal distributed variables. P-values ≤0.05 were considered significant.

## Results

### Clinical control

We defined clinical control as unchanged or improved neurological status after LINAC-SRS. After a median follow-up time of 11 years (range 5.3–22.1 years), two patients were free of symptoms and eight patients showed an improvement of one or more of their symptoms (hyp-/anacusis, pulsatile tinnitus, vertigo/dizziness, cephalgia, cranial nerve palsy, otalgia) after clinical examination (Table 3). In 12 patients the symptoms remained unchanged. Five patients died due to old age or other, not tumor- or treatment-related reasons 7.4, 9.6, 10.2, 12.4 and 13.1 years after LINAC-SRS. The last examination of these patients revealed a clinical improvement in two and an unchanged status in two patients. One patient developed a new permanent facial paresis grade II according to House & Brackmann six months after treatment (D_2_-tumor, tumor surface dose 20 Gy, treatment in 1991).

### Tumor control

**Fig 1:**
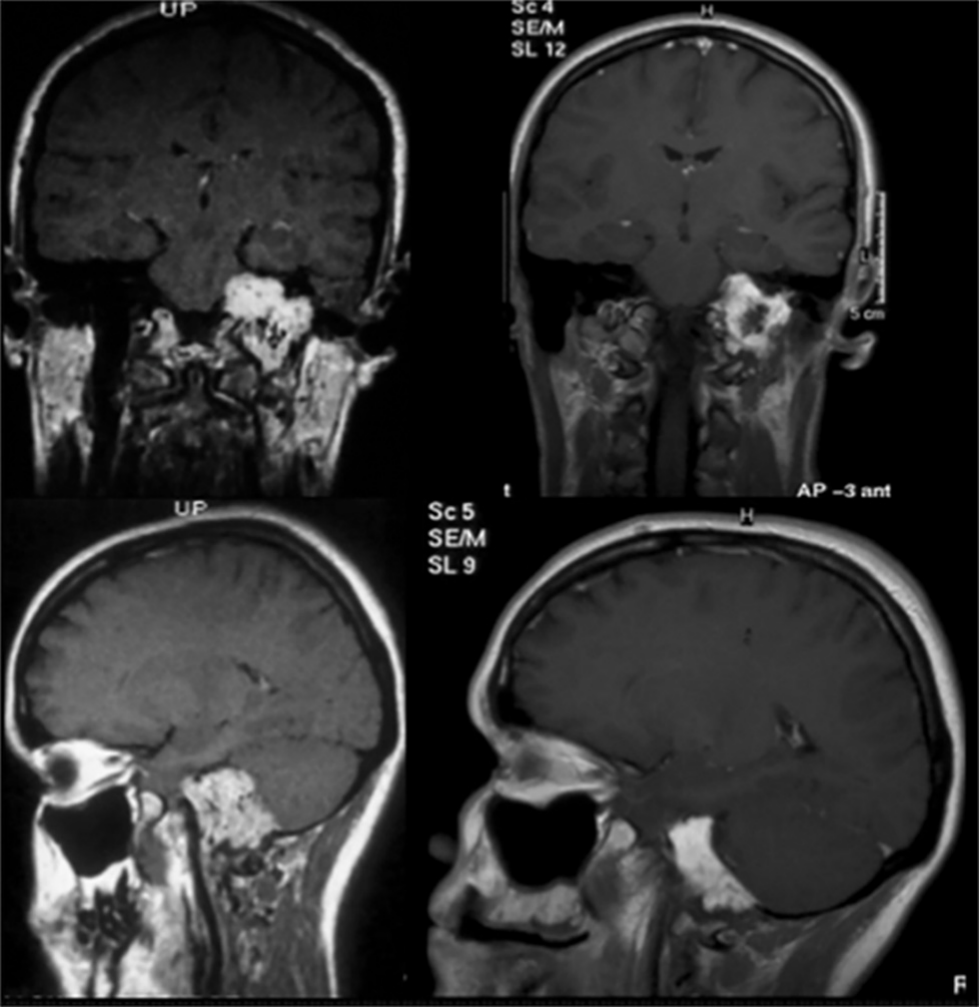
Follow-up T_1_-weighted, gadolinium enhanced MR-imaging of a 28-year old female with a left-sided GJT (D_2_) prior to LINAC-SRS (left) and 17.5 years later (right) showing a partial tumor remission.

**Fig 2:**
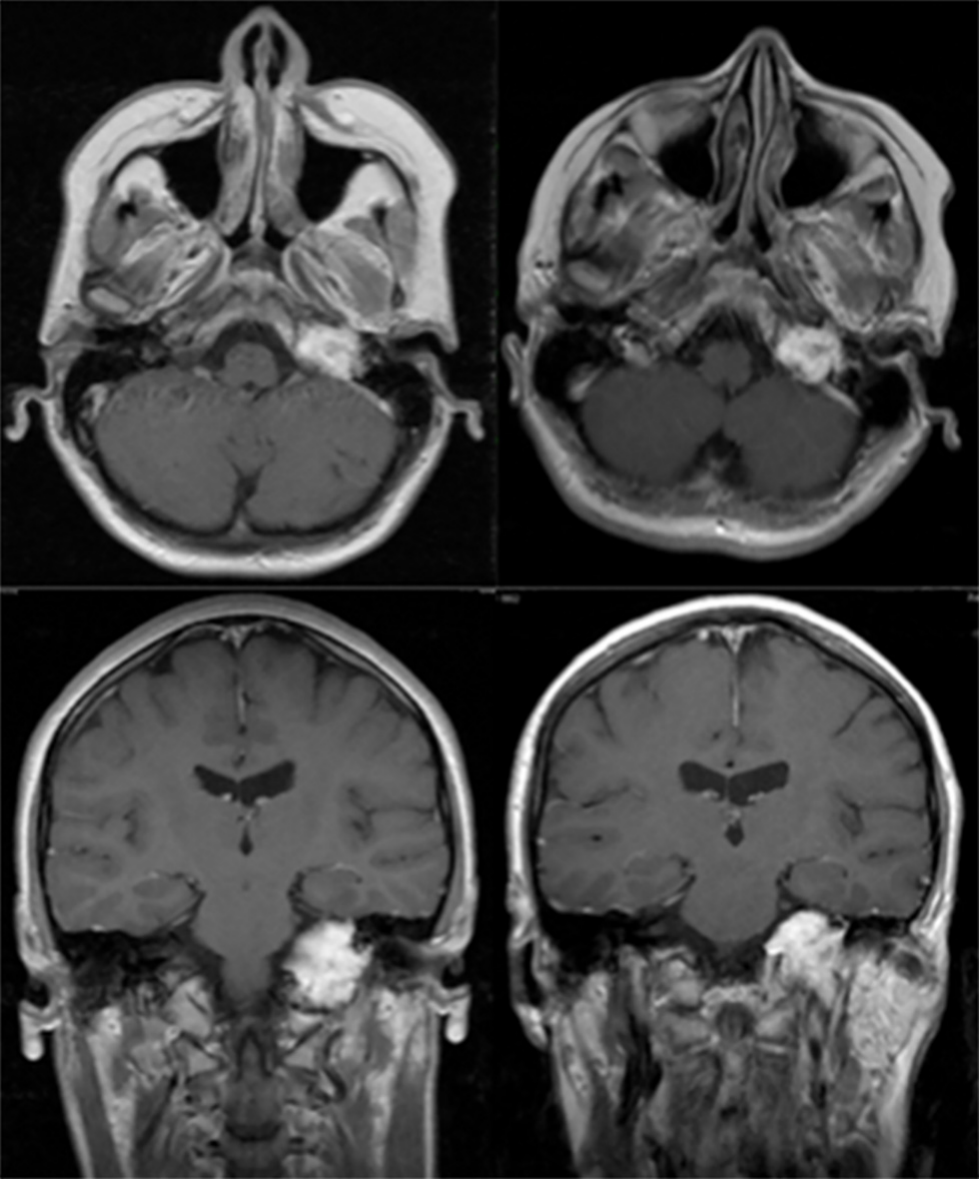
Follow-up T_1_-weighted, gadolinium enhanced MR-imaging of a 29-year old female with a left-sided GJT (D_1_) prior to LINAC-SRS (left) and 13.9 years later (right) showing a stable disease.

We defined tumor control as unchanged or decreased tumor volume after LINAC-RS assessed on MR-imaging. After a median radiological follow-up time of 9.6 years (range 5.1–19.1 years) 12 patients showed tumor shrinkage between 50.6–91% (partial response according to Macdonald) [20] and 15 patients showed tumor shrinkage between 33.3–49.4% (stable disease) displayed in Figs 1 and 2. We observed no tumor progression. Volumetric analysis exhibited median tumor shrinkage of 49.4% (mean 48.7%, range 33.3–91%), as displayed in Table 4. A radiation-induced secondary malignancy or meningiomas were not revealed in our series.

**Table 4:** Tumour response.

	median	mean	range
Tumor volume prior treatment (ml)	9.5	12.2	2.8–51
Tumor volume at last follow-up (ml)	4.7	6.0	0.9–21.7
Shrinkage (%)	49.4	48.7	33.3–91.0

## Discussion

In this study we have demonstrated the efficacy and safety of LINAC-RS for the treatment of GJTs in 27 patients either as primary or salvage therapy. Furthermore, we have presented a median long-term follow-up time of 11 years. To date, this study constitutes the largest series with the longest median follow-up time obtained at a single institution reported and published so far with this method.

In the natural history of GJTs there is an estimated annual median tumor growth of 0.83 mm and an estimated median tumor doubling time of 10 years [21]. Due to the rarity of this tumors there are limited data outlining the most appropriate therapeutic strategy consisting of microsurgical resection (with or without previous embolization), stereotactic radiosurgery (gamma knife, LINAC, cyber knife), conventional external fractionated irradiation or a combination of these therapeutic regimes.

The main treatment goal of this in general benign tumor should be to reduce morbidity rather than to increase survival. Hence, the risk of intervention should not be greater than the risk of the tumor’s natural course [11].

Microsurgery can offer a complete cure after gross total resection (GTR) but due to the high vascularization of GJTs and their location adjacent to critical structures a GTR can be challenging even in experienced hands. Outcomes reported in the literature following surgical resection are summarized in Table 5. GTR rates varied among the series between 85–96% [7, 12, 22–24]. Unfortunately, there was also a high rate of significant morbidity varying from 39–69% including cranial nerve weakness, hearing loss and dysphagia [7, 12, 22–24]. In addition to neurologic deficits other postsurgical complications that occur frequently are cerebrospinal fluid leaks, wound infections and meningitis. Mortality rates for patients with GJTs after microsurgical resection range between 1.7–6.4% [12, 13, 22]. The rates of local tumor control following microsurgery range between 83–90% (Table 5) with an averaging time to recurrence of 7 years [7, 12].

**Table 5:** Comparison of follow-up data after microsurgical resection.

Authors	Patients (n)	Median follow-up (months)	GTR (%)	Local control (%)	Morbidity^1^ (%)
Green et al. (1994)^[22]^	52	47	85	n.a.	n.a.
Jackson et al. (2001)^[12]^	176	54	90	90	59
Al-Mefty et al. (2002)^[7]^	28	38	86	86	39
Pareschi et al. (2003)^[24]^	37	59	96	n.a.	50
Sana et al. (2006)^[23]^	53	180	91	83	69

Abbreviations: GTR, gross total resection; n.a., not available

^1^mainly cranial nerve weakness, hearing loss, dysphagia

External fractionated radiotherapy (EBRT) as another treatment option for inoperable GJTs shows local tumor control rates between 85–94% [25–28] but has been criticized for the high radiation exposure to normal healthy tissue. Large fields have been irradiated including a significant safety margin to compensate immobilization and setup errors [17]. But it seems that serious late side effects caused by EBRT can be avoided if stereotactic fractionated radiotherapy is applied [16]. Thus, this method is a valuable tool for treating patients with large GJT’s not suitable for radiosurgery.

A more precise radiation technique represents stereotactic radiosurgery (SRS). This single-session and precise applied highly focused percutaneous irradiation allows to irradiate high single doses to GJTs while preserving adjacent healthy tissue due to a steep dose fall-off outside the target volume [29, 30]. The vast majority of SRS series report on results after gamma knife radiosurgery (GKRS). A total of 203 patients were treated with this technique within nine studies [4, 9, 31–37]. Follow-up ranged from 20–86.4 months showing a clinical improvement in 50% of patients and a permanent morbidity rate in less than 5%. Tumor size reduction has been reported in 11–50% of cases and a recurrence rate of 6%.

Much less data are available on treatment results after LINAC-SRS. Thirty-eight patients across four studies were evaluated showing comparable results to those in the GKRS series (Table 6) [17, 38–40]. But the introduction of the μMLC for LINAC-SRS allows a more homogeneous irradiation even of complex shaped tumor configurations by using only one irradiation field. That means dose inhomogeneities caused by field overlap, so called “hot spots”, that is common for EBRT or GKRS are eliminated. Thus, radiation-induced side effects are minimized.

**Table 6:** Comparison of follow-up data after LINAC-SRS.

Authors	Patients (n)	f/u^1^ (mths)	Tu Vol^1^ (ml)	Dose^1^ ^,^ ^2^ (Gy)	Clin Imp n (%)	Tu Shrin n (%)	Rec n (%)	s/e temp/perm (%)
Feigenberg et al. (2002)^[38]^	5	27	10.8	15	2 (40)	none	2 (40)	20/none
Maarouf et al. (2003)^[17]^	12	48	12.2	15	3 (25)	8 (67)	none	none/8
Lim et al. (2004)^[39]^	13^3^	60	3.0	20	n.a.	2 (15.4)	none	15.4/none
Poznanovic et al. (2006)^[40]^	8	16	7.3	15	7 (87.5)	2 (25)	none	25/none
Own study	27	148	12.2	15	12 (44.4)	12 (44.4)^4^	none	none/3.7

Abbreviations: f/u, follow-up; mths, months; Tu Vol, tumor volume; ml, milliliter, Gy, Gray; Clin Imp, clinical improvement; n.a., not available; Tu Shrin, tumor shrinkage; Rec, recurrences; s/e, side effects; temp, temporary; perm, permanent

^1^median values

^2^Tumor surface dose

^3^eight patients treated with cyberknife

^4^partial response according to Macdonald [20]

However, concerns have been raised regarding the development of radiation-induced second malignancies when treating benign tumors especially in young people. In the literature there are three cases described in connection with the irradiation of GJTs [41–43]. In one case published in 1967 a fibrosarcoma occurred 25 years after a local radium therapy [41]. The other two case reports published in 1979 and 1993 describe an anaplastic astrocytoma eight years after EBRT [43] and a low-grade fibrosarcoma 15 years after EBRT [42]. In our series we’ve not observed any treatment-induced second malignancies throughout the follow-up time.

Due to our promising long-term results beside a significant lower rate of morbidity compared to microsurgical series we consider LINAC-SRS as an alternative treatment for GJTs. Furthermore, one should consider that all treated tumors in our series corresponded to a high Fisch grade (D_1_ or D_2_) so that an inherent bias might be present and results could be better when treating GJTs of all grades.

## Supporting Information

S1 File German Data Protection Legislation, English Version. (PDF)
